# Resolution improvement of brain PET images using prior information from MRI: clinical application on refractory epilepsy

**DOI:** 10.1186/2197-7364-2-S1-A72

**Published:** 2015-05-18

**Authors:** Jesus Silva-Rodríguez, Charalampos Tsoumpas, Pablo Aguiar, Julia Cortes, Jesus Lopez Urdaneta

**Affiliations:** Instituto de Investigaciones Sanitarias (IDIS), Santiago de Compostela, Spain; University of Leeds, Leeds, UK; Nuclear Medicine Department, University Hospital (CHUS), Santiago de Compostela, Spain

An important counterpart of clinical Positron Emission Tomography (PET) for early diagnosis of neurological diseases is its low resolution. This is particularly important when evaluating diseases related to small hypometabolisms such as epilepsy. The last years, new hybrid systems combining PET with Magnetic Resonance (MR) has been increasingly used for several different clinical applications. One of the advantages of MR is the production of high spatial resolution images and a potential application of PET-MR imaging is the improvement of PET resolution using MR information. A potential advantage of resolution recovery of PET images is the enhancement of contrast delivering at the same time better detectability of small lesions or hypometabolic areas and more accurate quantification over these areas. Recently, Shidahara et al (2009) proposed a new method using wavelet transforms in order to produce PET images with higher resolution. We optimised Shidahara’s method (SFS-RR) to take into account possible shortcomings on the particular clinical datasets, and applied it to a group of patients diagnosed with refractory epilepsy. FDG-PET and MRI images were acquired sequentially and then co-registered using software tools. A complete evaluation of the PET/MR images was performed before and after the correction, including different parameters related with PET quantification, such as atlas-based metabolism asymmetry coefficients and Statistical Parametric Mapping results comparing to a database of 87 healthy subjects. Furthermore, an experienced physician analyzed the results of non-corrected and corrected images in order to evaluate improvements of detectability on a visual inspection. Clinical outcome was used as a gold standard. SFS-RR demonstrated to have a positive impact on clinical diagnosis of small hypometabolisms. New lesions were detected providing additional clinically relevant information on the visual inspection. SPM sensitivity for the detection of small lesions was increased from 70% to 90%.

